# Automatic wide complex tachycardia differentiation using mathematically synthesized vectorcardiogram signals

**DOI:** 10.1111/anec.12890

**Published:** 2021-09-25

**Authors:** Anthony H. Kashou, Sarah LoCoco, Trevon D. McGill, Christopher M. Evenson, Abhishek J. Deshmukh, David O. Hodge, Daniel H. Cooper, Sandeep S. Sodhi, Phillip S. Cuculich, Samuel J. Asirvatham, Peter A. Noseworthy, Christopher V. DeSimone, Adam M. May

**Affiliations:** ^1^ Department of Medicine Mayo Clinic Rochester Minnesota USA; ^2^ Division of Cardiovascular Diseases Department of Medicine Washington University School of Medicine in St. Louis St. Louis Missouri USA; ^3^ Department of Health Sciences Research Mayo Clinic Rochester Minnesota USA; ^4^ Department of Cardiovascular Diseases Mayo Clinic Rochester Minnesota USA

**Keywords:** electrocardiogram, supraventricular tachycardia, ventricular tachycardia, wide complex tachycardia

## Abstract

**Background:**

Automated wide complex tachycardia (WCT) differentiation into ventricular tachycardia (VT) and supraventricular wide complex tachycardia (SWCT) may be accomplished using novel calculations that quantify the extent of mean electrical vector changes between the WCT and baseline electrocardiogram (ECG). At present, it is unknown whether quantifying mean electrical vector changes within three orthogonal vectorcardiogram (VCG) leads (X, Y, and Z leads) can improve automated VT and SWCT classification.

**Methods:**

A derivation cohort of paired WCT and baseline ECGs was used to derive five logistic regression models: (i) one novel WCT differentiation model (i.e., VCG Model), (ii) three previously developed WCT differentiation models (i.e., WCT Formula, VT Prediction Model, and WCT Formula II), and (iii) one “all‐inclusive” model (i.e., Hybrid Model). A separate validation cohort of paired WCT and baseline ECGs was used to trial and compare each model's performance.

**Results:**

The VCG Model, composed of WCT QRS duration, baseline QRS duration, absolute change in QRS duration, X‐lead QRS amplitude change, Y‐lead QRS amplitude change, and Z‐lead QRS amplitude change, demonstrated effective WCT differentiation (area under the curve [AUC] 0.94) for the derivation cohort. For the validation cohort, the diagnostic performance of the VCG Model (AUC 0.94) was similar to that achieved by the WCT Formula (AUC 0.95), VT Prediction Model (AUC 0.91), WCT Formula II (AUC 0.94), and Hybrid Model (AUC 0.95).

**Conclusion:**

Custom calculations derived from mathematically synthesized VCG signals may be used to formulate an effective means to differentiate WCTs automatically.

## 1 INTRODUCTION

Twelve‐lead electrocardiogram (ECG) interpretation is the most practical means to non‐invasively differentiate wide complex tachycardias (WCTs) into ventricular tachycardia (VT) or supraventricular wide complex tachycardia (SWCT). Rigorous research spanning several decades has amassed an expansive arsenal of manual ECG interpretation methods (Kashou et al., 2021; Kashou, Noseworthy, et al., 2020), each relying upon the visual recognition of distinctive electrocardiographic features of VT and SWCT. Yet, despite the creation of numerous manual diagnostic criteria and algorithms, arriving at a correct and timely VT or SWCT diagnosis remains problematic.

Recent works (Kashou, DeSimone, Deshmukh, et al., 2020; May et al., 2019a, 2020) have introduced several novel automated methods capable of distinguishing VT and SWCT with high accuracy. Through the use of readily accessible ECG data routinely processed by computerized ECG interpretation software, automated methods (i.e., WCT Formula [2019] (May et al., 2019a), VT Prediction Model [2020] (May et al., 2020), and WCT Formula II [2020] (Kashou, DeSimone, Deshmukh, et al., 2020)) are able to deliver to clinicians an estimation of VT probability—one that is freely independent of ECG interpreter competency. By design, each automated approach makes use of paired WCT and baseline ECG data to deduce the magnitude of mean electrical vector changes contained within two ECG planes (i.e., frontal [limb leads] and horizontal [chest leads]). In the case of the WCT Formula (May et al., 2019a), the frontal and horizontal percent amplitude change (PAC) calculations are used to broadly quantify QRS amplitude (μV) changes in the frontal and horizontal planes, respectively. Similarly, the WCT Formula II (Kashou, DeSimone, Deshmukh, et al., 2020), comprised of frontal and horizontal percent time‐voltage area change (PTVAC) calculations, makes use of QRS time‐voltage area (TVA) (μV∙ms) changes in the frontal and horizontal planes, respectively. Alternatively, the VT Prediction Model (May et al., 2020) uses QRS axis (°) and T‐wave axis (°) change, both of which can be easily computed from standard computerized ECG measurements.

**FIGURE 1:**
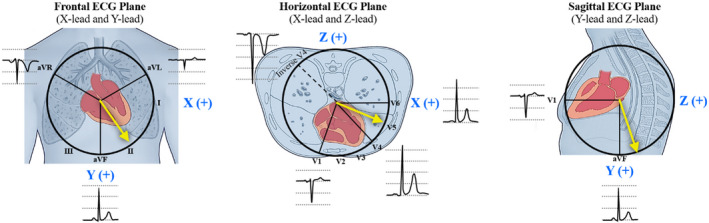
Mean ventricular depolarization vector in the frontal, horizontal, and sagittal ECG planes. The X‐lead appraises electrical changes from the patient's right to patient's left direction (frontal and horizontal ECG planes), the Y‐lead appraises electrical changes from the cranial‐to‐caudal direction (frontal and sagittal ECG planes), and the Z‐lead appraises electrical changes from the anterior‐to‐posterior direction (horizontal and sagittal ECG planes). Yellow arrows illustrate the archetypal direction and magnitude of a mean electrical vector of ventricular depolarization for a normal baseline ECG. The spatial orientation of each lead is depicted using blue (VCG leads) and black (standard 12‐lead ECG leads) font lettering. The directionality of VCG signal recordings is depicted by a “+” symbol (positive voltage—i.e., waveforms above the isoelectric baseline). ECG, electrocardiogram; VCG, vectorcardiogram

In this work, we sought to determine whether the quantification of QRS amplitude changes, between paired WCT and baseline ECGs, within three orthogonal vectorcardiogram (VCG) leads (i.e., X‐lead [patient's right to patient's left], Y‐lead [cranial‐to‐caudal], and Z‐lead [anterior‐to‐posterior]), may yield effective methods to differentiate WCTs automatically. Provided that QRS complex data from mathematically synthesized X‐, Y‐, and Z‐VCG leads can be configured to determine the mean electrical vector of depolarization across three spatial planes (i.e., frontal plane [X and Y leads], horizontal plane [X and Z leads], and sagittal plane [Y and Z leads]) (Figure 1), we hypothesized that QRS amplitude changes of mathematically synthesized VCG leads may offer a more robust means of quantifying changes in the mean electrical vector of depolarization and enable more accurate WCT differentiation.

## 2 METHODS

### 2.1 Study design

In this study, we formulated and trialed two novel logistic regression models (i.e., VCG Model and Hybrid Model) comprised of custom computations from mathematically synthesized VCG signals derived from paired WCT and subsequent baseline ECGs. The diagnostic performance of each model was directly compared to other previously described automated WCT differentiation models, namely the WCT Formula (May et al., 2019a), VT Prediction Model (May et al., 2020), and WCT Formula II (Kashou, DeSimone, Deshmukh, et al., 2020).

First, a derivation cohort of paired WCT and baseline ECGs was used to derive (i.e., VCG Model) and re‐derive (i.e., WCT Formula, VT Prediction Model, and WCT Formula II) automated logistic regression models. Concurrently, all variables which comprise the WCT Formula, VT Prediction Model, WCT Formula II, and VCG Model were collectively integrated to formulate the Hybrid Model. Second, all five logistic regression models were trialed on a separate validation cohort of paired WCT and baseline ECGs. The diagnostic performance metrics (i.e., accuracy, sensitivity, specificity, and area under the curve [AUC]) achieved by each model were directly compared.

Patient data acquisition and analysis was approved by the Mayo Clinic Institutional Review Board. Clinical and electrocardiographic data from patients of the derivation and validation cohorts were previously examined and described in prior works (Kashou, DeSimone, Hodge, et al., 2020; May et al., 2019b).

### 2.2 Electrocardiogram selection

Wide complex tachycardia and baseline ECG pairs were recorded within clinical settings at the Mayo Clinic Rochester or Mayo Clinic Health System of South Eastern Minnesota between September 2011 and November 2016. Evaluated ECGs were standard 12‐lead recordings (paper speed: 25 mm/s and voltage calibration: 10 mm/mV) accessed from centralized data archives provided by a proprietary ECG interpretation software system (MUSE [GE Healthcare]). WCTs were required to satisfy standard WCT criteria (QRS duration ≥120 ms and ventricular rate ≥100 beats per minute) and possess an official ECG laboratory interpretation of (i) “ventricular tachycardia,” (ii) “supraventricular tachycardia,” or (iii) “wide complex tachycardia.” Baseline ECGs were defined as the first non‐WCT rhythm recorded *after* the WCT event. Polymorphic WCTs and WCTs demonstrating grossly irregular atrioventricular conduction (e.g., atrial fibrillation or atrial flutter with variable atrioventricular block) were excluded. ECGs demonstrating truncated WCTs (e.g., brief run of non‐sustained VT) occurring within a dominant baseline heart rhythm (e.g., normal sinus rhythm) were not evaluated. If a WCT did not have a baseline ECG or definitive clinical diagnosis established by the patient’s overseeing physician, it was excluded from further analysis.

### 2.3 Derivation and validation cohorts

ECG pairs were collected from patients presenting to the Mayo Clinic Rochester or Mayo Clinic Health System of South Eastern Minnesota between September 2011 and November 2016. Of the 597 ECG pairs evaluated, 400 and 197 ECG pairs were randomly assigned to the derivation and validation cohorts, respectively.

### 2.4 Official ECG laboratory diagnosis

Official ECG interpretation was completed by expert ECG interpreters, including 7 heart rhythm cardiologists and 14 non‐heart rhythm cardiologists.

### 2.5 Clinical diagnoses

Clinical diagnoses (i.e., VT or SWCT) were established by the patient's supervising physician. Physicians responsible for clinical diagnoses were stratified according to a subjective hierarchy of clinical expertise: (i) heart rhythm cardiologist, (ii) non‐heart rhythm cardiologist, and (iii) non‐cardiologist. All physicians responsible for clinical diagnoses had access to the official ECG interpretation diagnosis provided by the ECG laboratory.

### 2.6 ECG measurements

#### 2.6.1 Computerized ECG measurements

Standard computerized ECG measurements for WCT and baseline ECGs, including QRS duration (ms), QRS axis (°), and T‐wave axis (°), were generated by *GE Healthcare's* MUSE ECG interpretation software. Computerized QRS amplitude (μV) and TVA (time‐voltage area) (μV∙ms) measurements of waveforms above (*r*/*R* and *r'*/*R’*) and below (*q*/*QS*, *s*/*S*, and *s'*/*S′*) the isoelectric baseline were automatically derived from the dominant QRS complex template of select ECG leads (i.e., aVR, aVL, aVF, V1, V4, and V6). Only amplitude and TVA measurements representative of QRS complex waveforms were analyzed.

#### 2.6.2 Manual VCG measurements

Three mathematically synthesized VCG signals (i.e., X, Y, and Z leads) were automatically generated by *GE Healthcare's* MUSE ECG interpretation software package. VCG signal QRS amplitude (μV) measurements of waveforms above (*r*/*R* and *r'*/*R’*) and below (*q*/*QS*, *s*/*S*, and *s'*/*S′*) the isoelectric baseline were directly measured (using electronic calipers provided by the MUSE ECG interpretation software package) by the first author (K.A.H.), who was blinded to patients’ clinical characteristics and final rhythm diagnosis (VT or SWCT). Special attention was made to exclude pacing stimuli (i.e., “pacing spikes”) from QRS complex amplitude measurements.

### 2.7 ECG parameters

#### 2.7.1 QRS duration change (ms)

QRS duration change denotes the absolute difference in QRS duration (ms) measurements between paired WCT and baseline ECGs.

#### 2.7.2 QRS axis change (°)

QRS axis change is the absolute difference in the frontal plane QRS axis (°) between paired WCT and baseline ECGs.

#### 2.7.3 T‐wave axis change (°)

T‐wave axis change represents the absolute difference in the frontal plane T‐wave axis (°) between paired WCT and baseline ECGs.

#### 2.7.4 Frontal and horizontal percent amplitude change (%)

Frontal and horizontal PACs are quantifiable measures of QRS amplitude change between paired WCT and baseline ECG recordings (Figures S1 and S2). They are derived from computerized QRS waveform (*q*/*QS*, *r*/*R*, *s*/*S*, *r'*/*R’*, and *s'*/*S*′) amplitude (μV) measurements from corresponding ECG leads of the frontal (aVR, aVL, aVF) and horizontal (V1, V4, V6) ECG planes, respectively.

#### 2.7.5 Frontal and horizontal percent time‐voltage area change (%)

Frontal and horizontal PTVACs are quantifiable measures of the degree of change in QRS TVA between paired WCT and baseline ECGs (Figures S3 and S4). They are derived from QRS complex waveform (*q*/*QS*, *r*/*R*, *s*/*S*, *r'*/*R’*, and *s'*/*S’*) TVA measurements from specific ECG leads within the corresponding frontal (aVR, aVL, aVF) or horizontal (V1, V4, V6) ECG planes, respectively.

### 2.8 VCG parameters

#### 2.8.1 X‐, Y‐, and Z‐lead QRS amplitude change (%)

The X‐, Y‐, and Z‐lead QRS amplitude change represents the comparative change in QRS amplitude between paired WCT and baseline ECGs within three separate orthogonally oriented VCG leads: X‐lead (patient's right to patient's left), Y‐lead (cranial‐to‐caudal), and Z‐lead (anterior‐to‐posterior), respectively (Figure 1). Unlike frontal and horizontal PAC or PTVAC, which can both be calculated from automated computerized QRS waveform measurements of standard ECG leads (aVR, aVL, aVF, V1, V4, and V6), X‐, Y‐, and Z‐lead QRS amplitude change calculations are derived from manual QRS waveform measurements of mathematically synthesized VCG signals generated by computerized ECG interpretation software. The mathematical procedure necessary to calculate X‐, Y‐, and Z‐lead QRS amplitude change is presented in Figure S5.

### 2.9 Logistic regression models

#### 2.9.1 WCT formula, VT prediction model, and WCT formula II

Prior work has described the logistic regression model structure of the WCT Formula (May et al., 2019a, 2019b), VT Prediction Model (Kashou, DeSimone, Hodge, et al., 2020; May et al., 2020), and WCT Formula II (Kashou, DeSimone, Deshmukh, et al., 2020). In general, each model uses independent VT predictors to derive an automatic estimation of VT probability (0.00%–99.99%). The logistic regression structure of the WCT Formula, VT Prediction Model, and WCT Formula II are shown in Figures S6–S8.

#### 2.9.2 VCG model

By design, the VCG Model integrates measured and calculated ECG data from paired WCT and baseline ECGs to deliver an unambiguous estimation of VT probability (0.00%–99.99%) using independent VT predictors synchronously weighted according to their influence on the binary classification of VT or SWCT. The logistic regression structure of the VCG Model is shown below:

$$X_{\beta }=\beta_{0}+\beta_{1}X_{1}+\beta_{2}X_{2}+\beta_{3}X_{3}+\beta_{4}X_{4}+\beta_{5}X_{5}+\beta_{6}X_{6}=\text{Ln}\left(\frac{P}{1‐P}\right)$$

$$X_{\beta }=‐12.4466\;+\;(0.009548)\;(X‐\text{lead}\;\text{QRS}\;\text{amplitude}\;\text{change})+(0.017794)\;(Y‐\text{lead}\;\text{QRS}\;\text{amplitude}\;\text{change})+(0.010624)\;(Z‐\text{lead}\;\text{QRS}\;\text{amplitude}\;\text{change})\;+\;(0.029447)\;(\text{WCT}\;\text{QRS}\;\text{duration})\;+\;(0.025475)\;(\text{Baseline}\;\text{QRS}\;\text{duration})\;+\;(0.039523)\;(\text{Absolute}\;\text{QRS}\;\text{duration}\;\text{change})=\text{Ln}\left(\frac{P}{1‐P}\right)$$

$$P=\left(\frac{e^{X_{\beta }}}{1\;+\;e^{X_{\beta }}}\right)$$

The VCG Model incorporates six parameters directly accessed or calculated from paired WCT and baseline ECG data: two standard ECG measurements (i.e., WCT QRS duration [ms] and baseline QRS duration [ms]), one arithmetic calculation of standard ECG measurements (i.e., QRS duration change [ms]), and three novel computations derived from mathematically synthesized VCG signals (i.e., X‐, Y‐, and Z‐lead QRS amplitude change [%]). Each of the six parameters (X*x*) are apportioned a beta coefficient (β*x*) according to their influence on binary classification. The “constant” term (β_0_) denotes the *y*‐intercept for the least‐squares regression line. The weighted sum predictor (X_β_) and VT probability (P) may be calculated after integrating VT predictor (X*x*) values derived from WCT and baseline ECG data.

#### 2.9.3 Hybrid model

Constituent variables comprising the WCT Formula, VT Prediction Model, WCT Formula II, and VCG Model were combined to build an “all‐inclusive” logistic regression model, which we refer to as the Hybrid Model. The Hybrid Model includes 12 variables: WCT QRS duration (ms), baseline QRS duration (ms), QRS duration change (ms), QRS axis change (°), T‐wave axis change (°), frontal PAC (%), horizontal PAC (%), frontal PTVAC (%), horizontal PTVAC (%), X‐lead QRS amplitude change (%), Y‐lead QRS amplitude change (%), and Z‐lead QRS amplitude change (%). A summary of the logistic regression structure of the Hybrid Model is shown in Figure S9.

### 2.10 Statistical analysis

Categorical variables were compared using chi‐square tests. Wilcoxon rank‐sum tests were used to compare continuous variables. In order to ensure satisfactory model comparisons, all logistic regression models were originally formulated or re‐derived using the same collection of ECG pairs comprising the derivation cohort. Thereafter, each model was trialed separately on the same validation cohort. Previously described logistic regression models, including the WCT Formula, VT Prediction Model, and WCT Formula II, were composed of the same variables as defined by the original works introducing each model (Kashou, DeSimone, Deshmukh, et al., 2020; May et al., 2019a, 2020). Paired ECGs of the validation cohort were assigned estimated VT probabilities (0.00%–99.99%) by all five logistic regression models. Binary rhythm classification (VT or SWCT) was rendered according to a pre‐specified VT probability partition of 50% (i.e., VT  ≥ 50% and SWCT  < 50%). Performance metrics (i.e., accuracy, sensitivity, specificity, positive predictive value [PPV], and negative predictive value [NPV]) for each model were assessed according to their agreement with the overseeing physician's clinical diagnosis. AUC was used to summarize overall diagnostic performance. Comparison of the fit between the statistical models was completed using a Delong test. A two‐tailed *p*‐value of <.05 was considered statistically significant. Statistical analyses were completed using SAS version 9.4 (SAS Institute).

## 3 RESULTS

### 3.1 Part I: Derivation of logistic regression models

#### 3.1.1 Derivation cohort

The derivation cohort included 400 paired WCT (185 VT, 215 SWCT) and baseline ECGs from 309 patients. Clinical diagnosis and ECG laboratory interpretation data are shown in Table S1. The majority (86.0%) of clinical diagnoses (VT or SWCT) were established by heart rhythm or non‐heart rhythm cardiologists. Two‐hundred and three out of the 400 (50.8%) VT or SWCT clinical diagnoses were established among patients having a corroborating electrophysiology procedure or implanted intra‐cardiac device (e.g., pacemaker).

Patient characteristics of VT and SWCT groups are described in Table S2. The VT group included more ECG pairs from patients with coronary artery disease, prior myocardial infarction, ongoing antiarrhythmic drug use, ischemic cardiomyopathy, and implanted implantable cardioverter‐defibrillator (ICD). The SWCT group included more ECG pairs from patients with an implanted pacemaker. The VT group comprised more patients with a severely depressed (≤30%) left ventricular ejection fraction (LVEF). Conversely, the SWCT group included more patients with a preserved (≥50%) LVEF. Baseline ECGs with ventricular pacing were more common in the VT group, whereas preexisting bundle branch block was more frequent in the SWCT group.

#### 3.1.2 ECG parameters

Paired ECGs in the VT group expressed greater WCT QRS duration, QRS duration change, QRS axis change, frontal PAC, horizontal PAC, frontal PTVAC, horizontal PTVAC, X‐lead QRS amplitude change, Y‐lead QRS amplitude change, and Z‐lead QRS amplitude change (Table 1).

**TABLE 1:** Electrocardiographic parameters

	WCT (*n* = 400)	SWCT (*n* = 215)	VT (*n* = 185)	*p*‐Value
WCT QRS duration (ms)	159.2 (31.2)	143.1 (17.9)	177.8 (32.9)	<.001
Baseline QRS duration (ms)	140.0 (34.1)	136.1 (23.5)	144.5 (43.0)	.180
Absolute QRS duration change (ms)	30.5 (31.2)	16.7 (17.8)	46.5 (35.5)	<.001
Change in QRS axis (°)	56.5 (57.2)	26.1 (34.1)	91.8 (58.4)	<.001
Change in T‐wave axis (°)	64.9 (56.7)	41.3 (42.9)	92.4 (58.6)	<.001
Frontal PAC (%)	79.4 (73.4)	37.8 (28.7)	127.6 (79.7)	<.001
Horizontal PAC (%)	78.1 (59.2)	42.6 (27.2)	119.3 (59.6)	<.001
Frontal PTVAC (%)	136.4 (152.1)	56.2 (51.0)	229.6 (175.7)	<.001
Horizontal PTVAC (%)	119.7 (121.1)	60.3 (51.0)	188.7 (141.0)	<.001
X‐lead QRS amplitude change (%)	74.8 (77.8)	45.6 (48.2)	108.6 (90.9)	<.001
Y‐lead QRS amplitude change (%)	91.3 (104.8)	44.9 (35.3)	145.1 (130.2)	<.001
Z‐lead QRS amplitude change (%)	92.4 (107.6)	49.9 (57.7)	141.7 (129.2)	<.001

Standard deviation is in parentheses.

Abbreviations: PAC, percent amplitude change; PTVAC, percent time voltage area change; SWCT, supraventricular wide complex tachycardia; VT, ventricular tachycardia; WCT, wide complex tachycardia.

#### 3.1.3 WCT formula, VT prediction model, and WCT formula II derivation

Logistic regression model derivation, using previously established model parameters for the WCT Formula, VT Prediction Model, and WCT Formula II, yielded excellent WCT differentiation with an AUC of 0.95, 0.91, and 0.94, respectively.

#### 3.1.4 VCG model derivation

The novel VCG Model composed of independent parameters, including WCT QRS duration (*p* < .001), baseline QRS duration (*p* = .007), absolute change in QRS duration (*p* < .001), X‐lead QRS amplitude change (*p* < .001), Y‐lead QRS amplitude change (*p* < .001), and Z‐lead QRS amplitude change (*p* < .001), demonstrated effective WCT differentiation (AUC 0.94) for the derivation cohort.

#### 3.1.5 Hybrid Model derivation

The “all‐inclusive” Hybrid Model composed of 12 unified parameters, including WCT QRS duration (*p* = .005), baseline QRS duration (*p* = .102), QRS duration change (*p* = .419), QRS axis change (*p* = .961), T‐wave axis change (*p* = .419), frontal PAC (*p* = .036), horizontal PAC (*p* = .361), frontal PTVAC (*p* = .148), horizontal PTVAC (*p* < .001), X‐lead QRS amplitude change (*p* = .260), Y‐lead QRS amplitude change (*p* = .124), and Z‐lead QRS amplitude change (*p* = .082) achieved favorable WCT differentiation (AUC 0.95) for the derivation cohort.

### 3.2 Part II: Validation of logistic regression models

#### 3.2.1 Validation cohort

The validation cohort comprised 197 paired WCT (86 VT, 111 SWCT) and baseline ECGs from 173 patients. Clinical diagnosis and ECG laboratory interpretation data are shown in Table S3. The majority (84.7%) of VT and SWCT clinical diagnoses were established by heart rhythm or non‐heart rhythm cardiologists. Ninety‐eight out of 197 (49.7%) clinical diagnoses were established among patients having a corroborating electrophysiology procedure or implanted intra‐cardiac device.

Patient characteristics data of VT and SWCT groups are described in Table S4. Comparison of clinical diagnosis, ECG interpretation data, and patient characteristics between the derivation and validation cohorts is shown in Tables S5 and S6.

#### 3.2.2 Validation of diagnostic performance

**FIGURE 2:**
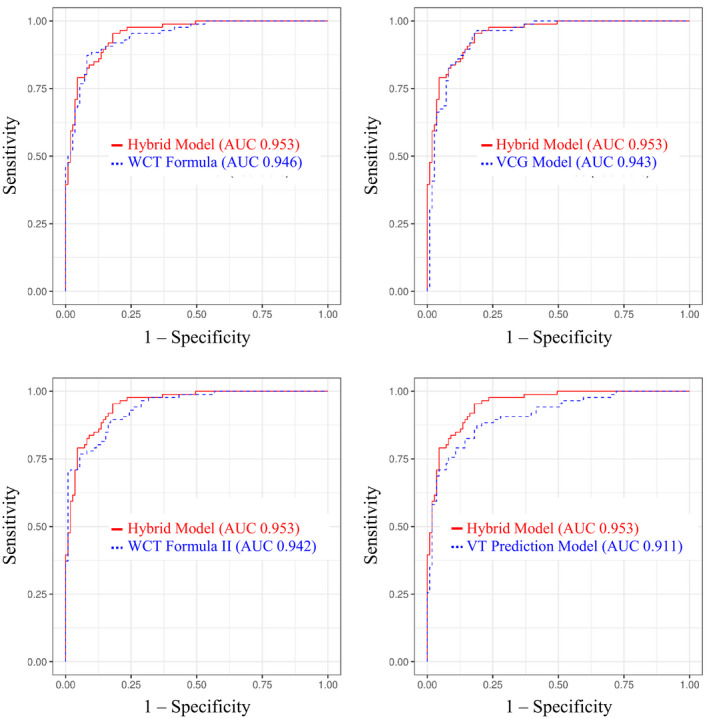
Comparison of the diagnostic performance of Hybrid Model against other WCT differentiation models. AUC, area under the receiver operating characteristic curve; VCG, vectorcardiogram; VT, ventricular tachycardia; WCT, wide complex tachycardia

Overall accuracy, sensitivity, specificity, PPV, NPV, and AUC of each logistic regression model is listed in Table 2. Comparisons of diagnostic performance metrics between each logistic regression model are presented in Table 3. Figure 2 illustrates diagnostic performance comparisons (i.e., AUC) between the Hybrid Model and all other logistic regression models.

**TABLE 2:** Logistic regression model diagnostic performance

	VCG Model	WCT Formula	WCT Formula II	VT Prediction Model	Hybrid Model
Accuracy (%)	87.8 (82.4–92.0)	87.8 (82.4–92.0)	87.8 (82.4–92.0)	83.3 (77.3–88.2)	86.8 (81.3–91.2)
Sensitivity (%)	83.7 (74.2–90.8)	82.6 (72.9–89.9)	83.7 (74.2–90.8)	75.6 (65.1–84.2)	80.2 (70.3–88.0)
Specificity (%)	91.0 (84.1–95.6)	91.9 (85.2–96.2)	91.0 (84.1–95.6)	89.2 (81.9–94.3)	91.9 (85.2–96.2)
PPV (%)	87.8 (78.7–94.0)	88.8 (79.7–94.7)	87.8 (78.7–94.0)	84.4 (74.4–91.7)	88.5 (79.2–94.6)
NPV (%)	87.8 (80.4–93.2)	87.2 (79.7–92.6)	87.8 (80.4–93.2)	82.5 (74.5–88.8)	85.7 (78.1–91.5)
AUC	0.94 (0.91–0.97)	0.95 (0.92–0.98)	0.94 (0.91–0.97)	0.91 (0.87–0.95)	0.95 (0.93–0.98)

Summary of logistic regression model performance metrics. Overall accuracy, sensitivity, specificity, positive predictive value, and negative predictive value rendered according to a pre‐specified VT probability partition of 50% (i.e., VT  ≥ 50% and SWCT < 50%). Numbers in parentheses are 95% confidence intervals.

Abbreviations: AUC, area under the curve; NPV, negative predictive value; PPV, positive predictive value.

**TABLE 3:** Comparison of logistic regression model performance

	Accuracy	Sensitivity	Specificity	AUC
Hybrid Model vs. WCT Formula	0.81	0.75	1.0	0.53
Hybrid Model vs. WCT Formula II	0.81	0.58	1.0	0.14
Hybrid Model vs. VCG Model	0.81	0.58	1.0	0.28
Hybrid Model vs. VT Prediction Model	0.15	0.29	0.51	0.01^*^
WCT Formula vs. WCT Formula II	1.0	1.0	1.0	0.80
WCT Formula vs. VCG Model	1.0	1.0	1.0	0.86
WCT Formula vs. VT Prediction Model	0.05	0.11	0.45	0.02^*^
WCT Formula II vs. VCG Model	1.0	1.0	1.0	0.98
WCT Formula II vs. VT Prediction Model	0.08	0.12	0.68	0.04^*^
VCG Model vs. VT Prediction Model	0.08	0.07	0.75	0.09

Summary of logistic regression model comparisons. Values depicted are *p* values (*p* value <.05 was considered statistically significant [*]).

Abbreviations: AUC, area under the curve; VT, ventricular tachycardia; WCT, wide complex tachycardia.

The VCG Model achieved effective VT and SWCT differentiation (AUC 0.94; confidence interval [CI] 0.91–0.97). When adopting a 50% VT probability partition for rhythm adjudication (i.e., VT  ≥ 50% and SWCT  < 50%), the VCG Model achieved an overall accuracy, sensitivity, and specificity of 87.8%, 83.7%, and 91.0%, respectively. Diagnostic performance indices of the VCG Model were similar to that achieved by the WCT Formula, VT Prediction Model, and WCT Formula II.

The Hybrid Model yielded effective WCT differentiation (AUC 0.95; CI 0.93–0.98). When applying a 50% VT probability partition for diagnosis (i.e., VT  ≥ 50% and SWCT  < 50%), the Hybrid Model yielded an overall accuracy, sensitivity, and specificity of 86.8%, 80.2%, and 91.9%. Overall diagnostic performance (i.e., AUC) of the Hybrid Model outperformed the VT Prediction Model but was otherwise comparable to that achieved by the WCT Formula, WCT Formula II, and VCG Model.

In addition, other model comparisons revealed that the WCT Formula and WCT Formula II demonstrated superior diagnostic performance compared to the VT Prediction Model. Otherwise, no other statistically significant differences in model performance were observed.

For each model, diagnostic performance did not differ among patients with or without a corroborating electrophysiology procedure or implantable intra‐cardiac device: WCT Formula (*p* = .67), WCT Formula II (*p* = .64), VT Prediction Model (*p* = .08), VCG Model (*p* = .64), and Hybrid Model (*p* = .98).

## 4 DISCUSSION

In this work, we sought to determine whether the quantification of QRS amplitude changes within three orthogonal VCG leads (X, Y, and Z leads) can be used to create an effective means for automated WCT classification. We demonstrated that accurate WCT differentiation may be achieved by novel logistic regression models (i.e., VCG Model and Hybrid Model) comprised of custom computations from mathematically synthesized VCG signals derived from paired WCT and baseline ECGs. We found VCG Model's performance was comparable to other high‐performing automated WCT differentiation models, but its novel constituents (i.e., X‐lead QRS amplitude change, Y‐lead QRS amplitude change, and Z‐lead QRS amplitude change) did not make an iterative improvement in WCT differentiation accuracy when unified with other established WCT differentiation parameters as part of the Hybrid Model.

### 4.1 Diagnostic performance

#### 4.1.1 VCG Model

Our study results showed that the VCG Model implementation yielded effective WCT differentiation (AUC 0.94) for WCTs expected to be encountered in clinical practice. Overall performance was similar whether or not the VCG Model was applied to patients who were diagnosed by the traditional “gold standard” methods, including rhythm classification based on the results of electrophysiology procedures or analysis of intra‐cardiac device (i.e., pacemaker or ICD) recordings. The use of a pre‐specified 50% VT probability cut‐point for WCT classification (i.e., VT  ≥ 50% and SWCT < 50%) yielded favorable diagnostic indices, including strong overall accuracy with high diagnostic sensitivity and specificity for VT. Moreover, VCG Model's performance was comparable to be other high‐performing WCT differentiation approaches, including the WCT Formula (AUC 0.95), VT prediction Model (AUC 0.91), and WCT Formula II (AUC 0.94).

#### 4.1.2 Hybrid Model

The Hybrid Model similarly demonstrated exceptional diagnostic performance when implemented on the validation cohort (AUC 0.95). Again, like other automated WCT differentiation models, the Hybrid Model's performance did not significantly differ among patients without and without electrophysiology procedures or an implantable intra‐cardiac device. Upon using a pre‐specified 50% VT probability cut‐point (i.e., VT ≥ 50% and SWCT < 50%) for WCT classification, the Hybrid Model achieved strong overall accuracy with favorable diagnostic sensitivity and specificity for VT. Notably, the Hybrid Model outperformed the VT Prediction Model (AUC 0.91), but demonstrated similar diagnostic performance with the WCT Formula (AUC 0.95), WCT Formula II (AUC 0.94), and VCG Model (AUC 0.94).

### 4.2 X‐, Y‐, and Z‐lead QRS amplitude change

In prior works (Kashou, DeSimone, Deshmukh, et al., 2020; May et al., 2019a), we demonstrated that the quantification of QRS amplitude or TVA changes in specific leads of the frontal (aVR, aVL, aVF) and horizontal (V1, V4, V6) ECG planes enable accurate WCT differentiation. The underlying conceptual basis for these observations is that VT, which may originate and spread from any location within the right or left ventricles, categorically demonstrates immense "electrical freedom" compared to SWCT, which ordinarily depolarizes the ventricular myocardium in a manner prescribed by the native His‐Purkinje network (Evenson et al., 2021). Consequently, VT may express a virtually unlimited diversity of distinct QRS complexes dissimilar from those present on the respective baseline ECG. Correspondingly, VT will commonly demonstrate greater changes to the electrical vector of depolarization than SWCT upon WCT onset or offset. The few notable exceptions to this concept include VTs that rapidly engage (e.g., fascicular VT) or characteristically make use of the heart's His‐Purkinje network (e.g., bundle branch reentry). In contrast, SWCTs are expected to ordinarily demonstrate a more constrained range of mean electrical vectors and limited number of electrocardiographically distinct QRS complexes compared to the patient's baseline ECG. In rarer circumstances, marked mean electrical vector and QRS complex changes may arise from SWCTs that develop from impulse propagation using atrioventricular accessory pathways (i.e., pre‐excitation).

**FIGURE 3:**
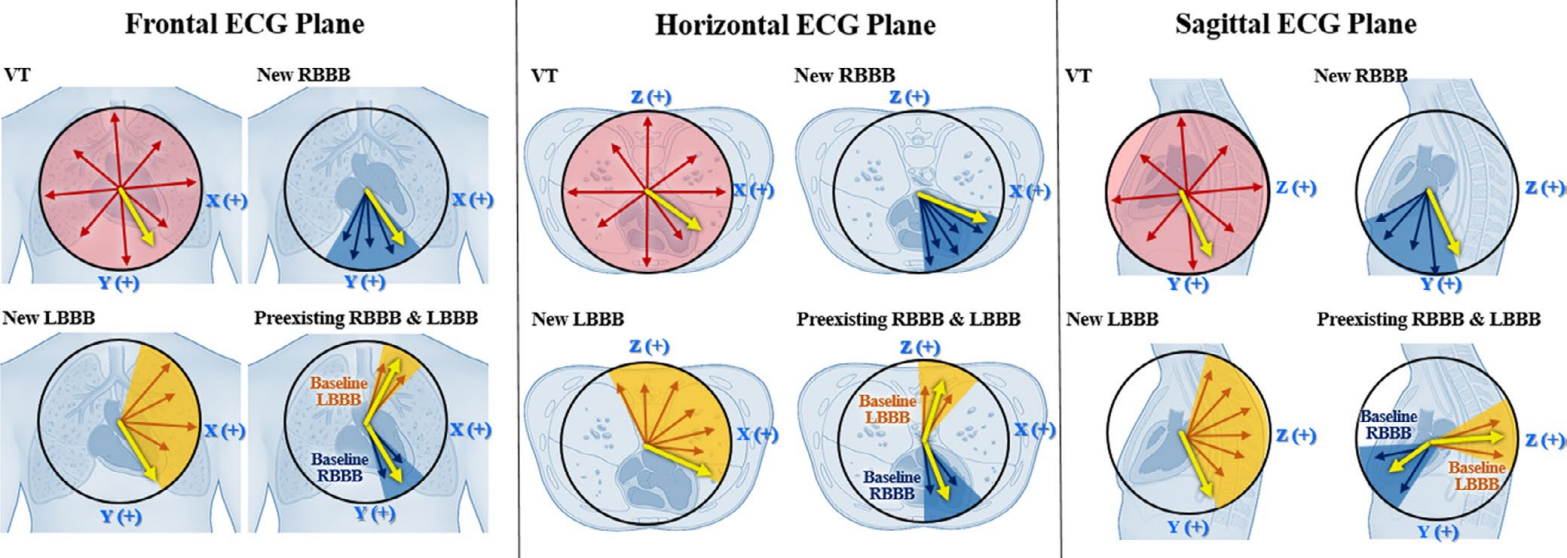
Mean electrical vector changes in the frontal, horizontal, and sagittal ECG planes. Summary of expected changes to the mean electrical vector of ventricular depolarization following WCT initiation within three orthogonal ECG planes (i.e., frontal, horizontal, and sagittal). Displayed arrows represent mean electrical vectors for ventricular depolarization. The directional orientation of individual arrows expresses the mean electrical axis of ventricular depolarization (i.e., QRS axis). The heavy yellow arrows denote the baseline heart rhythm's mean electrical vector for ventricular depolarization. Color‐shaded regions and arrows denote the expected range of mean electrical vectors after WCT onset. The spatial orientation of VCG leads is depicted with blue font lettering. The directionality of ventricular depolarization captured by VCG leads is portrayed by a “+” symbol (positive voltage—i.e., waveforms above the isoelectric baseline). The X‐lead appraises electrical changes from the patient's right to patient left direction (frontal and horizontal ECG planes), the Y‐lead appraises electrical changes from the cranial‐to‐caudal direction (frontal and sagittal ECG planes), and the Z‐lead appraises electrical changes from the anterior‐to‐posterior direction (horizontal and sagittal ECG planes). Panels show various examples of expected changes in the mean electrical vector of depolarization that occur upon WCT initiation within each orthogonal ECG plane (i.e., frontal, horizontal, and sagittal). VT exhibits a virtually unlimited range of potential mean electrical vectors. SWCTs due to new RBBB or new LBBB exhibit a relatively constrained range of possible mean electrical vectors. SWCTs arising from a preexisting RBBB or LBBB demonstrate minor changes to the mean electrical vector. ECG, electrocardiogram; LBBB, left bundle branch block; RBBB, right bundle branch block SWCT, supraventricular wide complex tachycardia; VT, ventricular tachycardia; WCT, wide complex tachycardia

Similar to other previously introduced variables (e.g., frontal PAC, horizontal PTVAC, and QRS axis change) (Kashou, DeSimone, Deshmukh, et al., 2020; May et al., 2019a, 2020), X‐, Y‐, and Z‐lead QRS amplitude change are designed to quantify the extent of mean electrical vector changes that occur upon WCT onset or offset. However, unlike previously described diagnostic variables, the collective evaluation of X‐, Y‐, and Z‐lead QRS amplitude change enables the direct quantification of mean electrical vector changes occurring in three, instead of two, spatial planes (i.e., patient's right to patient's left [X‐lead], cranial‐to‐caudal [Y‐lead], and anterior‐to‐posterior [Z‐lead]) (Figure 3). Therefore, we hypothesized X‐, Y‐, and Z‐lead QRS amplitude changes may offer a more robust means to quantify changes in the mean electrical vector of depolarization, thereby enabling more accurate automated WCT differentiation.

We report X‐, Y‐, and Z‐lead QRS amplitude change are strong independent VT predictors—VT generally generates greater X‐, Y‐, and Z‐lead QRS amplitude change than SWCT. As such, X‐, Y‐, and Z‐lead QRS amplitude change independently contributed to WCT differentiation as part of the VCG Model. However, once X‐, Y‐, and Z‐lead QRS amplitude change were unified with other highly interrelated variables (e.g., frontal and horizontal PAC) as part of the all‐inclusive Hybrid Model, these parameters no longer maintained diagnostic singularity. Moreover, automated models that incorporated X‐, Y‐, and Z‐lead QRS amplitude change did not demonstrate superior diagnostic performance compared to previously introduced automated models, except in the case of the Hybrid Model outperforming the VT Prediction Model.

### 4.3 Prospective applications and future directions

The VCG Model and Hybrid Model join other recently introduced automated approaches (i.e., WCT Formula [2019] (May et al., 2019a), VT Prediction Model [2020] (May et al., 2020), and WCT Formula II [2020] (Kashou, DeSimone, Deshmukh, et al., 2020)) that are designed to leverage the diagnostic value of WCT and baseline ECG comparison and provide clinicians an impartial estimation VT probability through computerized ECG interpretation software. Similar to other recently introduced WCT differentiation models, the VCG Model and Hybrid Model may be of practical clinical use for medical providers upon their successful integration into computerized ECG interpretation software. Candidate computerized ECG interpretation software programs include those that are able to (i) identify, discriminate, and measure QRS complex and T‐wave waveforms accurately and reliably, and (ii) simultaneously process computerized ECG data from the WCT itself and the baseline ECG recorded (and archived) *before or after* the WCT event. Once automated algorithms are fully integrated into computerized ECG interpretation software, ECG interpreters and clinicians would be able to assimilate automatically delivered VT probability estimates (e.g., 90% VT probability) with other meaningful diagnostic information (e.g., patient history of ischemic heart disease, WCT demonstrating a “northwest axis,” or VT diagnosis reached by the 3rd step of the Brugada algorithm [i.e., presence of atrioventricular dissociation]).

Despite what would be an incredible diagnostic advantage, a glaring limitation of the VCG Model, Hybrid Model, and other recently introduced automated approaches is that they require the use of a recorded and digitally archived baseline ECG for their application. In circumstances where WCT patients present without a baseline ECG, clinicians and ECG interpreters would have to temporarily rely on manual WCT differentiation methods until the patient’s baseline ECG is acquired.

In future works, the customized variables that make up the VCG Model and other automated WCT differentiation models may be integrated with additional computerized ECG measurements or yet to be formulated diagnostic determinants to bolster diagnostic performance. Additionally, customized variables, which comprise already described logistic regression models, could be used by more sophisticated modeling techniques (e.g., artificial neural networks) more apt to decipher meaningful non‐linear and non‐parametric relationships.

### 4.4 Study limitations

Study limitations were comprehensively described in prior works (Kashou, DeSimone, Deshmukh, et al., 2020; May et al., 2019a, 2020). However, there are two important limitations that warrant special attention. First, our study evaluated any clinically encountered WCT (i.e., “all‐comers”) that was formally diagnosed by the patient's overseeing physician. As a result, this analysis did not exclude patients who did not undergo an electrophysiology procedure or did not have an implanted intra‐cardiac device (e.g., ICD or pacemaker). Although we found model performance did not differ among patients with or without an accompanying electrophysiology procedure or implanted intra‐cardiac device, we must acknowledge that a substantial proportion of VT or SWCT diagnoses were not established by the traditional “gold standard.” Furthermore, this WCT selection process precluded a detailed assessment of automated model performance among various SWCT (e.g., SWCT due to bystander conduction over various accessory pathways) and VT (e.g., fascicular VT) subtypes. Nevertheless, by not excluding WCT tracings lacking a corroborating electrophysiology procedure or implanted intra‐cardiac device, our analysis counters the patient selection biases created by only evaluating a proportionally smaller subgroup of WCTs seen in clinical practice. Second, the diagnostic performance of automated models was not directly compared with traditional manual WCT differentiation approaches (Brugada et al., 1991; Chen et al., 2019; Jastrzebski et al., 2017; Pava et al., 2010; Vereckei et al., 2008). Additional research will be necessary to determine whether automated models exhibit diagnostic superiority over conventional manual interpretation methods.

## 5 CONCLUSION

Custom calculations derived from mathematically synthesized VCG signals may be used to formulate accurate diagnostic models to differentiate VT and SWCT. The VCG Model and Hybrid Model, which incorporate customized VCG signal calculations, performed similarly well when compared to other high‐performing automated WCT differentiation models and may be incorporated into existing ECG interpretation software systems.

## CONFLICT OF INTEREST

This technology was disclosed to Mayo Clinic Ventures who possesses intellectual property rights.

## ETHICAL APPROVAL

Study protocol complied with the Declaration of Helsinki and was approved by the Mayo Clinic Institutional Review Board.

## AUTHOR CONTRIBUTIONS

Anthony H. Kashou: Investigation, Methodology, Writing ‐ original draft, Writing ‐ review & editing. Sarah LoCoco: Writing ‐ review & editing. Trevon D. McGill: Writing ‐ review & editing. Christopher M. Evenson: Writing ‐ review & editing. Abhishek J. Deshmukh: Funding acquisition, Investigation, Methodology, Writing ‐ review & editing. David O. Hodge: Formal analysis, Methodology, Writing ‐ review & editing. Daniel H. Cooper: Writing ‐ review & editing. Sandeep S. Sodhi: Writing ‐ review & editing. Phillip S. Cuculich: Writing ‐ review & editing. Samuel J. Asirvatham: Writing ‐ review & editing. Peter A. Noseworthy: Investigation, Methodology, Funding acquisition, Writing ‐ review & editing. Christopher V. DeSimone: Funding acquisition, Investigation, Methodology, Writing ‐ review & editing. Adam M. May: Conceptualization, Data curation, Funding acquisition, Investigation, Methodology, Writing ‐ original draft, Project administration, Writing ‐ review & editing.

## Supporting information

Supplementary Material

Table S1‐6

## Data Availability

The data that support the findings of this study are available from the corresponding author upon reasonable request.
