# Supplementary material for: Automatic wide complex tachycardia differentiation using mathematically synthesized vectorcardiogram signals
Source: Ann Noninvasive Electrocardiol. 2021 Sep 25;27(1):e12890. doi: 10.1111/anec.12890 (PMC8739609; doi:10.1111/anec.12890)
Supplement: Supplementary file 1 — Supplementary Material [file ANEC-27-e12890-s002.pdf]

Supplementary Figure S1

$PA = (+)\,Amplitude = (r \text{ or } R) + (r' \text{ or } R')(uV)$

$NA = (-)\,Amplitude = (QS \text{ or } q) + (s \text{ or } S) + (s' \text{ or } S')(uV)$

APC = Absolute Positive Change (uV)

ANC = Absolute Negative Change (uV)

TAC = Total Amplitude Change (uV)

TBA = Total Baseline Amplitude (uV)

Frontal BA = Frontal Plane Baseline Amplitude (uV)

Frontal ACC = Frontal Plane Absolute Amplitude Change (uV)

Frontal PAC = Frontal Plane Percent Amplitude Change (%)

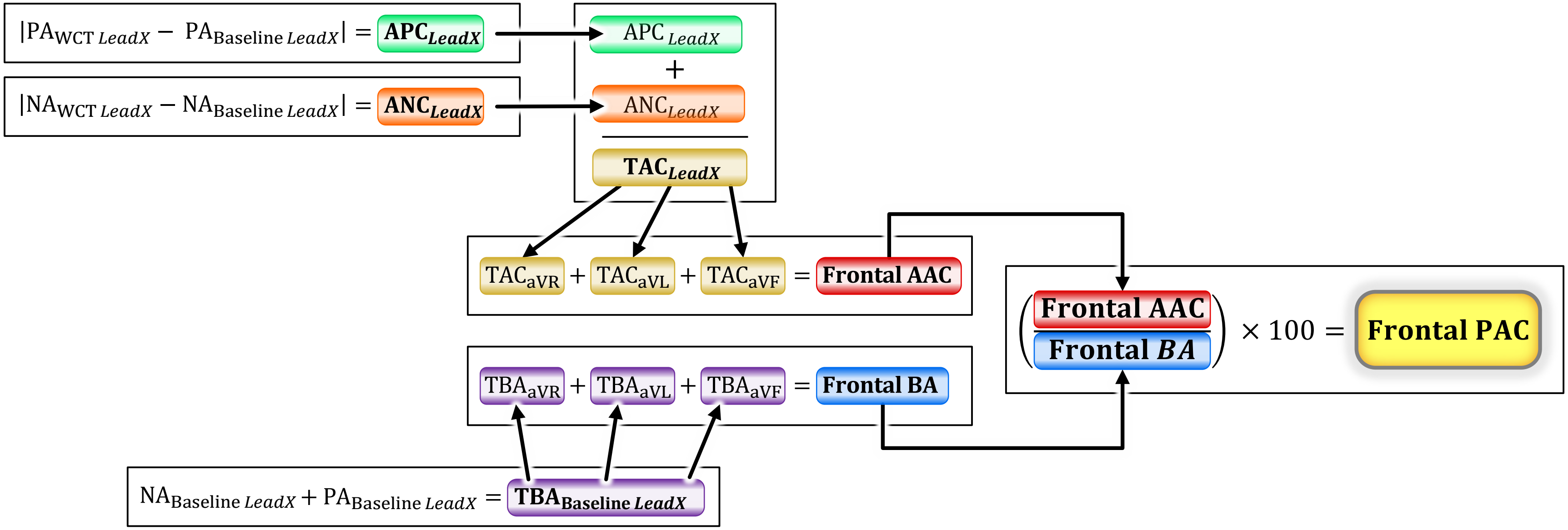

The frontal PAC (%) calculation is composed of measured QRS waveform amplitudes (μV) derived from select ECG leads within the frontal plane. *LeadX* denotes individual ECG leads within the frontal (aVR, aVL, aVF) ECG plane. Positive Amplitude (PA) is the sum of measured QRS waveform amplitudes above the isoelectric baseline (r/R and r'/R') in a single ECG lead. Negative Amplitude (NA) is the sum of measured QRS waveform amplitudes below the isoelectric baseline (q/QS, s/S, and s'/S') in a single ECG lead. Total Baseline Amplitude (TBA) is the sum of PA and NA within individual ECG leads of the baseline ECG. Baseline Amplitude (BA) is the summation of TBAs from select ECG leads in the frontal (aVR, aVL, aVF) ECG plane. Absolute Positive Change (APC) and Absolute Negative Change (ANC) are an individual ECG lead's absolute QRS amplitude change above and below the isoelectric baseline, respectively. Total Amplitude Change (TAC) is the sum of APC and ANC within an individual ECG lead. Absolute Amplitude Change (AAC) is the combined sum of TACs from select ECG leads of the frontal (aVR, aVL, aVF) ECG plane. Percent Amplitude Change (PAC) is the percent ratio of AAC to BA.

©2018 MFMER | 3726661-1

Supplementary Figure S2

$$\textbf{PA} = \textbf{(+) Amplitude} = (\text{r or R}) + (\text{r}' \text{ or R}')(\text{uV})$$
$$\textbf{NA} = \textbf{(−) Amplitude} = (\text{QS or q}) + (\text{s or S}) + (\text{s}' \text{ or S}')(\text{uV})$$
$$\textbf{APC} = \text{Absolute Positive Change (uV)}$$
$$\textbf{ANC} = \text{Absolute Negative Change (uV)}$$
$$\textbf{TAC} = \text{Total Amplitude Change (uV)}$$
$$\textbf{TBA} = \text{Total Baseline Amplitude (uV)}$$
$$\textbf{Horizontal BA} = \text{Horizontal Plane Baseline Amplitude (uV)}$$
$$\textbf{Horizontal ACC} = \text{Horizontal Plane Absolute Amplitude Change (uV)}$$
$$\textbf{Horizontal PAC} = \text{Horizontal Plane Percent Amplitude Change (\%)}$$

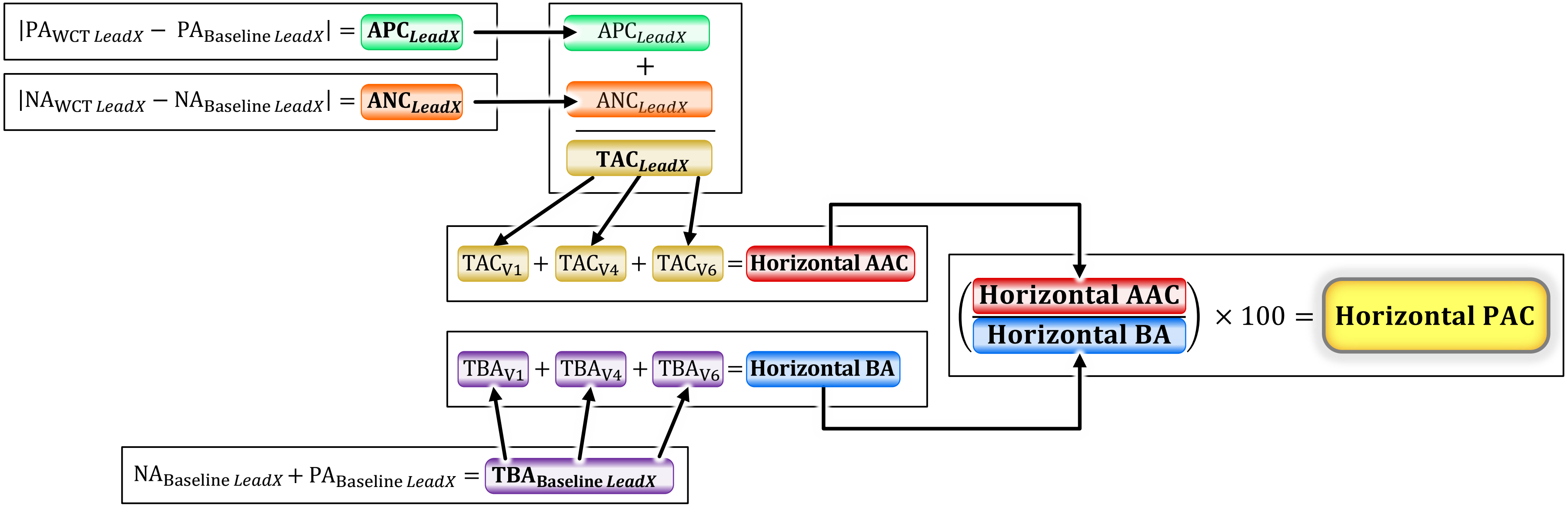

The horizontal PAC (%) calculation is composed of measured QRS waveform amplitudes (µV) derived from select ECG leads within the horizontal plane. *LeadX* denotes individual ECG leads within the horizontal (V1, V4, V6) ECG plane. Positive Amplitude (PA) is the sum of measured QRS waveform amplitudes above the isoelectric baseline (r/R and r'/R') in a single ECG lead. Negative Amplitude (NA) is the sum of measured QRS waveform amplitudes below the isoelectric baseline (q/QS, s/S, and s'/S') in a single ECG lead. Total Baseline Amplitude (TBA) is the sum of PA and NA within individual ECG leads of the baseline ECG. Baseline Amplitude (BA) is the summation of TBAs from select ECG leads in the horizontal (V1, V4, V6) ECG plane. Absolute Positive Change (APC) and Absolute Negative Change (ANC) are an individual ECG lead's absolute QRS amplitude change above and below the isoelectric baseline, respectively. Total Amplitude Change (TAC) is the sum of APC and ANC within an individual ECG lead. Absolute Amplitude Change (AAC) is the combined sum of TACs from select ECG leads of the horizontal (V1, V4, V6) ECG plane. Percent Amplitude Change (PAC) is the percent ratio of AAC to BA.

©2018 MFMER | 3726661-2

Supplementary Figure S3

PA = (+) Time−Voltage Area = (r or R)+(r′ or R′)(μV· ms)

NA = (−) Time−Voltage Area = (QS or q)+(s or S)+(s′ or S′)(μV· ms)

APC = Absolute Positive Change (μV· ms)

ANC = Absolute Negative Change (μV· ms)

TTVAC = Total Time−Voltage Area Change (μV· ms)

TBTVA = Total Baseline Time−Voltage Area (μV· ms)

Frontal BTVA = Frontal Plane Baseline Time−Voltage Area (μV· ms)

Frontal ATVAC = Frontal Plane Absolute Time−Voltage Area Change (μV· ms)

Frontal PTVAC = Frontal Plane Percent Time−Voltage Area Change (%)

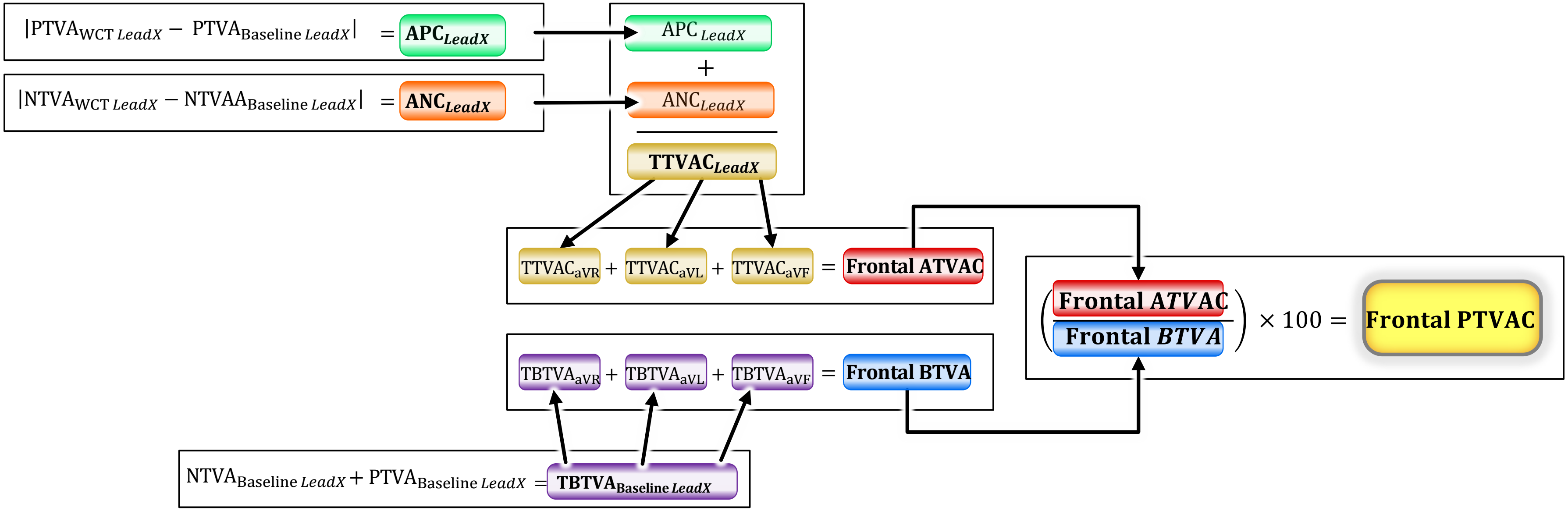

The frontal PTVAC (%) calculation is composed of measured QRS waveform time-voltage areas (μV· ms) derived from select ECG leads within the frontal plane. *LeadX* denotes individual ECG leads within the frontal (aVR, aVL, aVF) ECG plane. Positive Area (PA) is the sum of measured QRS waveform time-voltage areas above the isoelectric baseline (*r/R* and *r'/R'*) in a single ECG lead. Negative Area (NA) is the sum of measured QRS waveform time-voltage areas below the isoelectric baseline (*q/QS*, *s/S*, and *s'/S'*) in a single ECG lead. Total Baseline Time-Voltage Area (TBTVA) is the sum of PA and NA within individual ECG leads of the baseline ECG. Baseline Time-Voltage Area (BTVA) is the summation of TBTVAs from select ECG leads in the frontal (aVR, aVL, aVF) ECG plane. Absolute Positive Change (APC) and Absolute Negative Change (ANC) are an individual ECG lead's absolute QRS time-voltage area change above and below the isoelectric baseline, respectively. Total Time-Voltage Area Change (TTVAC) is the sum of APC and ANC within an individual ECG lead. Absolute Time-Voltage Area Change (ATVAC) is the combined sum of TTVACs from select ECG leads of the frontal (aVR, aVL, aVF) ECG planes. Percent Time-Voltage Area Change (PTVAC) is the percent ratio of ATVAC to BTVA.

©2018 MFMER | 3726661-3

Supplementary Figure S4

PA = (+) Time−Voltage Area = (r or R)+(r′ or R′)(μV· ms)

NA = (−) Time−Voltage Area = (QS or q)+(s or S)+(s′ or S′)(μV· ms)

APC = Absolute Positive Change (μV· ms)

ANC = Absolute Negative Change (μV· ms)

TTVAC = Total Time−Voltage Area Change (μV· ms)

TBTVA = Total Baseline Time−Voltage Area (μV· ms)

Horizontal BTVA = Horizontal Plane Baseline Time−Voltage Area (μV· ms)

Horizontal ATVAC = Horizontal Plane Absolute Time−Voltage Area Change (μV· ms)

HorizontalPTVAC = Horizontal Plane Percent Time−Voltage Area Change (%)

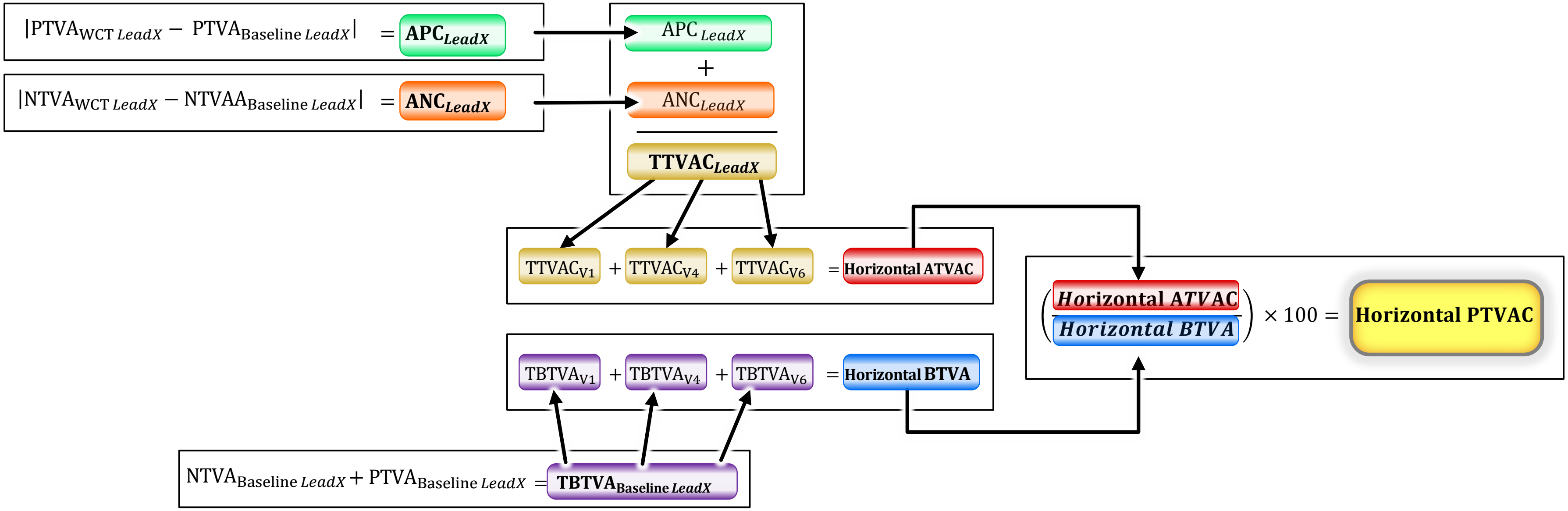

The horizontal PTVAC (%) calculation is composed of measured QRS waveform time-voltage areas (μV· ms) derived from select ECG leads within the horizontal plane. *LeadX* denotes individual ECG leads within the horizontal (V1, V4, V6) ECG plane. Positive Area (PA) is the sum of measured QRS waveform time-voltage areas above the isoelectric baseline (*r/R* and *r'/R'*) in a single ECG lead. Negative Area (NA) is the sum of measured QRS waveform time-voltage areas below the isoelectric baseline (*q/QS*, *s/S*, and *s'/S'*) in a single ECG lead. Total Baseline Time-Voltage Area (TBTVA) is the sum of PA and NA within individual ECG leads of the baseline ECG. Baseline Time-Voltage Area (BTVA) is the summation of TBTVAs from select ECG leads in the horizontal (V1, V4, V6) ECG plane. Absolute Positive Change (APC) and Absolute Negative Change (ANC) are an individual ECG lead's absolute QRS time-voltage area change above and below the isoelectric baseline, respectively. Total Time-Voltage Area Change (TTVAC) is the sum of APC and ANC within an individual ECG lead. Absolute Time-Voltage Area Change (ATVAC) is the combined sum of TTVACs from select ECG leads of the horizontal (V1, V4, V6) ECG plane. Percent Time-Voltage Area Change (PTVAC) is the percent ratio of ATVAC to BTVA.

©2018 MFMER | 3726661-4

Supplementary Figure S5

PA = (+) Amplitude = $(r \text{ or } R)+(r' \text{ or } R')$(uV)
NA = (−) Amplitude = $(QS \text{ or } q)+(s \text{ or } S)+(s' \text{ or } S')$(uV)
APC = Absolute Positive Change (uV)
ANC = Absolute Negative Change (uV)
TAC = Total Amplitude Change (uV)
BA = Baseline Amplitude (uV)

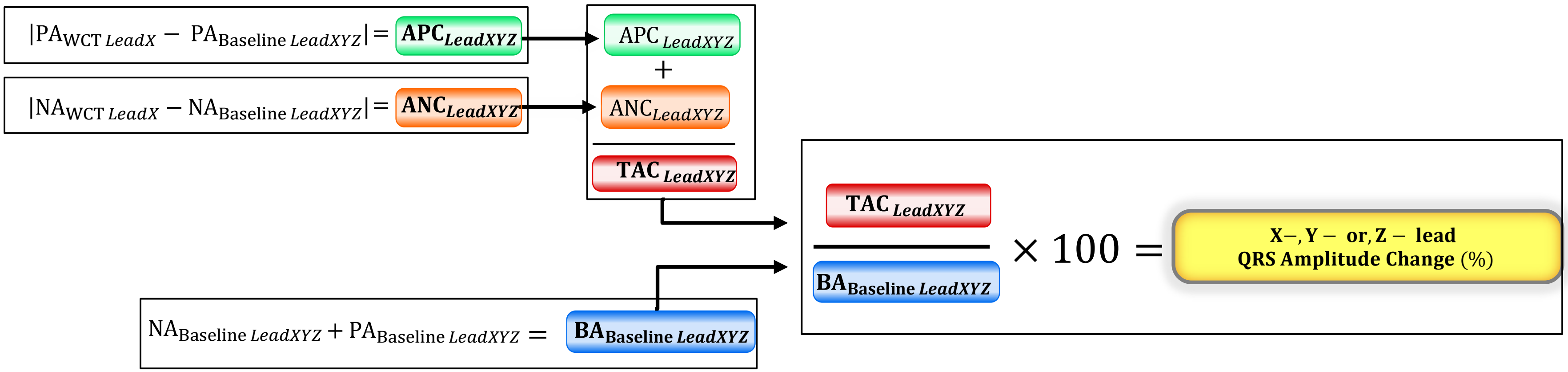

The X-,Y-, and Z- lead QRS amplitude change (%) calculations are composed of measured QRS waveform amplitudes (μV) derived from select mathematically synthesized VCG leads (X-,Y-, or Z- leads). *LeadXYZ* denotes any individual VCG lead: X-,Y-, or Z- leads. Positive Amplitude (PA) is the sum of measured QRS waveform amplitudes above the isoelectric baseline (r/R and r'/R') in a single VCG lead. Negative Amplitude (NA) is the sum of measured QRS waveform amplitudes below the isoelectric baseline (q/QS, s/S, and s'/S') in a single VCG lead. Total Baseline Amplitude (TBA) is the sum of PA and NA within an individual VCG lead derived from the baseline ECG. Baseline Amplitude (BA) is the summation of TBAs from individual VCG leads. Absolute Positive Change (APC) and Absolute Negative Change (ANC) are an individual VCG lead's absolute QRS amplitude change above and below the isoelectric baseline, respectively. Total Amplitude Change (TAC) is the sum of APC and ANC within an individual VCG lead. X-,Y-, or Z- lead QRS amplitude change (%) is the percent ratio of TAC to BA for individual VCG leads (X-,Y-, or Z- leads).

©2018 MFMER | 3726661-5

Supplementary Figure S6

WCT Formula

$$\mathbf{X}_{\boldsymbol{\beta}} = \beta_0 + \beta_1 X_1 + \beta_2 X_2 + \beta_3 X_3 = \mathrm{Ln}\left(\frac{P}{1-P}\right)$$

$$\mathbf{X}_{\boldsymbol{\beta}} = -15.89 + (0.068)(\text{WCT QRS duration}) + 0.030(\text{Frontal PAC}) + 0.041(\text{Horizontal PAC}) = \mathrm{Ln}\left(\frac{P}{1-P}\right)$$

$$\mathbf{P} = \left(\frac{e^{\mathbf{X}_{\boldsymbol{\beta}}}}{1+e^{\mathbf{X}_{\boldsymbol{\beta}}}}\right)$$

Logistic regression structure of the WCT Formula. VT predictors (X*x*) are assigned a beta coefficient (β*x*) according to their effect on the binary outcome (i.e., VT or SWCT). The “constant” term (β_0) represents the y-intercept for the least-squares regression line. The weighted sum predictor (X_β) or VT probability (P) is calculated after integrating VT predictor (X*x*) values derived from paired WCT and baseline ECG data. *ECG, electrocardiogram; SWCT, supraventricular tachycardia; VT, ventricular tachycardia; WCT, wide complex tachycardia.*

Supplementary Figure S7

VT Prediction Model

$$\mathbf{X_\beta} = \beta_0 + \beta_1 X_1 + \beta_2 X_2 + \beta_3 X_3 + \beta_4 X_4 = \text{Ln}\left(\frac{P}{1-P}\right)$$

$$\mathbf{X_\beta} = -10.785832 + (0.053543)(\text{WCT QRS duration}) + (0.013490)(\text{Absolute QRS duration change}) + (0.021582)(\text{QRS axis change}) + (0.011516)(\text{T axis change}) = \text{Ln}\left(\frac{P}{1-P}\right)$$

$$\mathbf{P} = \left(\frac{e^{\mathbf{X_\beta}}}{1+e^{\mathbf{X_\beta}}}\right)$$

Logistic regression structure of the VT Prediction Model. VT predictors (X*x*) are assigned a beta coefficient (β*x*) according to their effect on the binary outcome (i.e., VT or SWCT). The "constant" term (β_0) represents the y-intercept for the least-squares regression line. The weighted sum predictor (X_β) or VT probability (P) is calculated after integrating VT predictor (X*x*) values derived from paired WCT and baseline ECG data. *ECG, electrocardiogram; SWCT, supraventricular tachycardia; VT, ventricular tachycardia; WCT, wide complex tachycardia.*

Supplementary Figure S8

WCT Formula II

$$\mathbf{X}_{\boldsymbol{\beta}} = \beta_0 + \beta_1 X_1 + \beta_2 X_2 + \beta_3 X_3 + \beta_4 X_4 = \mathrm{Ln}\left(\frac{P}{1 - P}\right)$$

$$\mathbf{X}_{\boldsymbol{\beta}} = -14.504674 + (0.040987)(\text{Baseline QRS duration}) + (0.030295)(\text{WCT QRS duration}) + (0.019093)(\text{Frontal PTVAC}) + (0.015931)(\text{Horizontal PTVAC}) = \mathrm{Ln}\left(\frac{P}{1 - P}\right)$$

$$\mathbf{P} = \left(\frac{e^{\mathbf{X}_{\boldsymbol{\beta}}}}{1 + e^{\mathbf{X}_{\boldsymbol{\beta}}}}\right)$$

Logistic regression structure of the WCT Formula II. VT predictors (X*x*) are assigned a beta coefficient (β*x*) according to their effect on the binary outcome (i.e., VT or SWCT). The "constant" term (β_0) represents the y-intercept for the least-squares regression line. The weighted sum predictor (X_β) or VT probability (P) is calculated after integrating VT predictor (X*x*) values derived from paired WCT and baseline ECG data. *ECG, electrocardiogram; SWCT, supraventricular tachycardia; VT, ventricular tachycardia; WCT, wide complex tachycardia.*

Supplementary Figure S9

Hybrid Model

$$\mathbf{X_\beta} = \beta_0 + \beta_1 X_1 + \beta_2 X_2 + \beta_3 X_3 + \beta_4 X_4 + \beta_5 X_5 + \ldots \beta_x X_x = \mathrm{Ln}\left(\frac{P}{1-P}\right)$$

$$\mathbf{X_\beta} = \begin{matrix} -12.4466 \\ + (0.003464\ )\,(\text{X}-\text{lead QRS amplitude change}) \\ + (0.006413)(\text{Y}-\text{lead QRS amplitude change}) \\ + (0.004880)(\text{Z}-\text{lead QRS amplitude change}) \\ + (0.048018)(\text{WCT QRS duration}) \\ + (0.023242)(\text{Baseline QRS duration}) \\ + (0.014063)(\text{Absolute QRS duration change}) \\ + (-0.000283)(\text{QRS axis change}) \\ + (0.002706)(\text{T axis change}) \\ + (0.010027)(\text{Frontal PAC}) \\ + (0.038309)(\text{Horizontal PAC}) \\ + (0.008816)(\text{Frontal PTVAC}) \\ + (\text{-}0.004312)(\text{Horizontal PTVAC}) \end{matrix} = \mathrm{Ln}\left(\frac{P}{1-P}\right)$$

$$\mathbf{P} = \left(\frac{e^{\mathbf{X_\beta}}}{1+e^{\mathbf{X_\beta}}}\right)$$

Logistic regression structure of the Hybrid Model. VT predictors (X*x*) are assigned a beta coefficient (β*x*) according to their effect on the binary outcome (i.e., VT or SWCT). The “constant” term (β_0) represents the y-intercept for the least-squares regression line. The weighted sum predictor (X_β) or VT probability (P) is calculated after integrating VT predictor (X*x*) values derived from paired WCT and baseline ECG data. *ECG, electrocardiogram; SWCT, supraventricular tachycardia; VT, ventricular tachycardia; WCT, wide complex tachycardia.*
