# Supplementary material for: Automatic wide complex tachycardia differentiation using mathematically synthesized vectorcardiogram signals
Source: Ann Noninvasive Electrocardiol. 2021 Sep 25;27(1):e12890. doi: 10.1111/anec.12890 (PMC8739609; doi:10.1111/anec.12890)
Supplement: Supplementary file 2 — Table S1‐6 [file ANEC-27-e12890-s001.docx]

**SUPPLEMENTARY TABLES**

**Supplementary Table S1: Clinical and ECG laboratory diagnosis of derivation cohort.^[[1]](#footnote-1)^**

| Derivation Cohort | SWCT (n = 215) | VT (n = 185) | p-value |
| --- | --- | --- | --- |
| Diagnosing provider | | | |
| Heart rhythm cardiologists | 99 (46.0) | 166 (90.2) | <0.001 |
| Non-heart rhythm cardiologists | 65 (30.2) | 13 (7.1) |  |
| Non-cardiologists | 51 (23.7) | 5 (2.7) |  |
| ECG lab interpretation | | | |
| Definite VT | 10 (4.7) | 149 (81.0) | <0.001 |
| Probable VT | 11 (5.1) | 19 (10.3) |  |
| Definite SWCT | 169 (78.6) | 5 (2.7) |  |
| Probable SWCT | 10 (4.7) | 3 (1.6) |  |
| Undifferentiated | 15 (7.0) | 8 (4.3) |  |
| Time separation between WCT and Baseline ECG (hours) | | | |
| Mean (SD) | 292.2 (1344.9) | 160.1 (672.0) | 0.876 |
| Median | 7.0 | 8.3 |  |
| Time separation between WCT and Baseline ECG | | | |
| < 3 hours | 84 (39.1) | 75 (40.5) | 0.155 |
| 3 - 24 hours | 57 (26.5) | 44 (23.8) |  |
| 24 hours - 30 days | 54 (25.1) | 58 (31.4) |  |
| > 30 days | 20 (9.3) | 8 (4.3) |  |
| Electrophysiology procedure | | | |
| Yes | 39 (18.1) | 97 (52.4) | <0.001 |
| Implanted Device | | | |
| Yes | 30 (14.0) | 120 (64.9) | <0.001 |

| Derivation Cohort | SWCT (n = 215) | VT (n =185) | p-value |
| --- | --- | --- | --- |
| Age (years) | | | |
| Mean (SD) | 70 (14) | 67 (12) | 0.007 |
| Range | 18 - 97 | 27 - 90 |  |
| Gender | | | |
| Male | 144 (67.0) | 159 (85.9) | <0.001 |
| Female | 71 (33.0) | 26 (14.1) |  |
| Clinical Characteristics | | | |
| Coronary artery disease | 102 (47.4) | 127 (68.6) | <0.001 |
| Prior myocardial infarction | 59 (27.4) | 105 (56.8) | <0.001 |
| Prior cardiac surgery | 80 (37.2) | 81 (43.8) | 0.181 |
| Congenital heart disease | 13 (6.0) | 15 (8.1) | 0.420 |
| Anti-arrhythmic drug use | 31 (14.4) | 113 (61.1) | <0.001 |
| Ischemic cardiomyopathy | 31 (14.4) | 90 (48.6) | <0.001 |
| Non-ischemic cardiomyopathy | 52 (24.2) | 58 (31.4) | 0.110 |
| ICD | 13 (6.0) | 116 (62.7) | <0.001 |
| Pacemaker | 17 (7.9) | 4 (2.2) | 0.010 |
| Left Ventricular Ejection Fraction (%) | | | |
| LVEF (>= 50) | 125 (58.1) | 50 (27.0) | < 0.001 |
| LVEF (49 - 31) | 38 (17.7) | 61 (33.0) |  |
| LVEF (<= 30) | 44 (20.5) | 73 (39.5) |  |
| LVEF Unknown | 8 (3.7) | 1 (0.5) |  |
| Baseline ECG | | | |
| Baseline bundle branch block | 142 (66.0) | 28 (15.1) | <0.001 |
| Baseline ventricular pacing | 11 (5.1) | 75 (40.5) | <0.001 |

**Supplementary Table S2: Clinical characteristics of derivation cohort.^[[2]](#footnote-2)^**

**Supplementary Table S3: Clinical and ECG laboratory diagnosis of validation cohort.^[[3]](#footnote-3)^**

| Validation Cohort | SWCT (n = 111) | VT (n =86) | p-value |
| --- | --- | --- | --- |
| Age (years) | | | |
| Mean (SD) | 73 (15) | 64.0 (15) | <0.001 |
| Range | 19 - 98 | 31 - 86 |  |
| Gender | | | |
| Male | 68 (61.3) | 65 (75.6) | 0.033 |
| Female | 43 (38.7) | 21 (24.4) |  |
| Clinical Characteristics | | | |
| Coronary artery disease | 57 (51.4) | 59 (68.6) | 0.015 |
| Prior myocardial infarction | 34 (30.6) | 51 (59.3) | 0.0001 |
| Prior cardiac surgery | 42 (37.8) | 35 (40.7) | 0.683 |
| Congenital heart disease | 5 (4.5) | 4 (4.7) | 0.961 |
| Anti-arrhythmic drug use | 20 (18.0) | 51 (59.3) | <0.001 |
| Ischemic cardiomyopathy | 21 (18.9) | 47 (54.7) | <0.001 |
| Non-ischemic cardiomyopathy | 24 (21.6) | 31 (36.0) | 0.025 |
| AICD | 9 (8.1) | 59 (68.6) | <0.001 |
| Pacemaker | 10 (9.0) | 0 (0.0) | 0.004 |
| Left Ventricular Ejection Fraction (%) | | | |
| LVEF (>= 50) | 63 (56.8) | 18 (20.9) | <0.001 |
| LVEF (49 - 31) | 20 (18.0) | 24 (27.9) |  |
| LVEF (<= 30) | 22 (19.8) | 44 (51.2) |  |
| LVEF Unknown | 6 (5.4) | 0 (0.0) |  |
| Baseline ECG | | | |
| Baseline bundle branch block | 73 (65.8) | 10 (11.6) | <0.001 |
| Baseline ventricular pacing | 8 (7.2) | 34 (39.5) | <0.001 |

**Supplementary Table S4: Clinical characteristics of validation cohort.^[[4]](#footnote-4)^**

| Validation Cohort | SWCT (n = 111) | VT (n =86) | p-value |
| --- | --- | --- | --- |
| Diagnosing provider | | | |
| Heart rhythm cardiologists | 41 (36.9) | 79 (91.9) | <0.001 |
| Non-heart rhythm cardiologists | 43 (38.7) | 4 (4.7) |  |
| Non-cardiologists | 27 (24.3) | 3 (3.5) |  |
| ECG lab interpretation | | | |
| Definite VT | 0 (0.0) | 74 (86.0) | <0.001 |
| Probable VT | 5 (4.5) | 7 (8.1) |  |
| Definite SWCT | 94 (84.7) | 1 (1.2) |  |
| Probable SWCT | 6 (5.4) | 2 (2.3) |  |
| Undifferentiated | 6 (5.4) | 2 (2.3) |  |
| Time separation between WCT and Baseline ECG (hours) | | | |
| Mean (SD) | 561.7 (3255.8) | 163.3 (549.5) | 0.507 |
| Median | 5.4 | 6.0 |  |
| Time separation between WCT and Baseline ECG | | | |
| < 3 hours | 49 (44.1) | 35 (40.7) | 0.344 |
| 3 - 24 hours | 30 (27.0) | 19 (22.1) |  |
| 24 hours - 30 days | 24 (21.6) | 28 (32.6) |  |
| > 30 days | 8 (7.2) | 4 (4.7) |  |
| Electrophysiology procedure | | | |
| Yes | 11 (9.9) | 43 (50.0) | <0.001 |
| Implanted Device | | | |
| Yes | 19 (17.1) | 59 (68.6) | <0.001 |

|  | | Derivation Cohort (n = 400) | | Validation Cohort (n = 197 ) | | p-value |
| --- | --- | --- | --- | --- | --- | --- |
| Diagnosing Provider | | | | | | |
| Heart rhythm cardiologists | | 265 (66.4) | | 120 (60.9) | | 0.384 |
| Non-Heart rhythm cardiologists | | 78 (19.5) | | 47 (23.9) | |  |
| Non-cardiologists | | 56 (14.0) | | 30 (15.2) | |  |
| Time Separation between WCT and Baseline ECG (hours) | | | | | | |
| Mean (SD) | | 231.1 (1087.5) | | 387.8 (2473.7) | | 0.760 |
| Median | | 8.1 | | 5.5 | |  |
| Q1, Q3 | | 1.0, 47.0 | | 1.2, 43.0 | |  |
| Range | | (0.0 – 15093.9) | | (0.0 – 29800.2) | |  |
| Time Separation between WCT and Baseline ECG | | | | | | |
| < 3 hours | | 159 (39.8) | | 84 (42.6) | | 0.905 |
| 3 - 24 hours | | 101 (25.3) | | 49 (24.9) | |  |
| 1 - 30 days | | 112 (28.) | | 52 (26.4) | |  |
| >= 30 days | | 28 (7.0) | | 12 (6.1) | |  |
| ECG Lab Interpretation | | | | | | |
| Definite VT | | 159 (39.8) | | 74 (37.6) | | 0.707 |
| Probable VT | | 30 (7.5) | | 12 (6.1) | |  |
| Definite SWCT | | 174 (43.6) | | 95 (48.2) | |  |
| Probable SWCT | | 13 (3.3) | | 8 (4.1) | |  |
| Undifferentiated | | 23 (5.8) | | 8 (4.1) | |  |
| Electrophysiology Procedure | | | | | | |
| Yes | | 136 (34.0) | | 54 (27.4) | | 0.104 |
| Implantable Device | | | | | | |
| Yes | 150 (37.5) | | 78 (39.6) | | 0.621 | |

**Supplementary Table S5: Comparison of the clinical and ECG laboratory diagnosis from the derivation and validation cohorts.^[[5]](#footnote-5)^**

**Supplementary Table S6: Comparison of the patient characteristics inderivation and validation cohorts.^[[6]](#footnote-6)^**

|  | Derivation Cohort (n = 400) | Validation Cohort (n = 197 ) | p-value |
| --- | --- | --- | --- |
| Age (years) | | | |
| Mean (SD) | 68 (13) | 69 (16) | 0.276 |
| Range | 18 – 97 | 19 – 98 |  |
| Gender | | | |
| Male | 303 (75.8) | 133 (67.5) | 0.033 |
| Female | 64 (32.5) | 97 (24.3) |  |
|  | | | |
| Coronary artery disease | 229 (57.3) | 116 (58.9) | 0.704 |
| Prior myocardial infarction | 164 (41.0) | 85 (43.1) | 0.617 |
| Prior cardiac surgery | 161 (40.3) | 77 (39.1) | 0.785 |
| Congenital heart disease | 28 (7.0) | 9 (4.6) | 0.247 |
| Anti-arrhythmic drug use | 144 (36.0) | 71 (36.0) | 0.992 |
| Ischemic cardiomyopathy |  |  |  |
| Non-ischemic cardiomyopathy | 110 (27.5) | 55 (27.9) | 0.914 |
| ICD | 129 (32.3) | 68 (34.5) | 0.580 |
| Pacemaker | 21 (5.3) | 10 (5.1) | 0.928 |
| Left Ventricular Ejection Fraction (%) | | | |
| LVEF (>= 50) | 175 (43.8) | 81 (41.1) | 0.649 |
| LVEF (49 - 31) | 99 (24.8) | 44 (22.3) |  |
| LVEF (<= 30) | 117 (29.3) | 66 (33.5) |  |
| Unknown LVEF | 9 (2.3) | 6 (3.0) |  |
| Baseline ECG | | | |
| Baseline bundle branch block | 170 (42.5) | 83 (42.1) | 0.932 |
| Baseline ventricular pacing | 86 (21.5) | 42 (21.3) | 0.960 |

1. Numbers in parentheses are percent (%) of n or standard deviation. *Abbreviations: SD = standard deviation; SWCT = supraventricular wide complex tachycardia; VT = ventricular tachycardia.* [↑](#footnote-ref-1)
2. Numbers in parentheses are percent (%) of n or standard deviation. *Abbreviations: ICD = implantable cardioverter-defibrillator; LVEF = left ventricular ejection fraction; SD = standard deviation; SWCT = supraventricular wide complex tachycardia; VT = ventricular tachycardia.* [↑](#footnote-ref-2)
3. Numbers in parentheses are percent (%) of n or standard deviation. *Abbreviations: SD = standard deviation; SWCT = supraventricular wide complex tachycardia; VT = ventricular tachycardia.* [↑](#footnote-ref-3)
4. Numbers in parentheses are percent (%) of n or standard deviation. *Abbreviations: ICD = implantable cardioverter-defibrillator; LVEF = left ventricular ejection fraction; SD = standard deviation; SWCT = supraventricular wide complex tachycardia; VT = ventricular tachycardia.* [↑](#footnote-ref-4)
5. Numbers in parentheses are percent (%) of n or standard deviation. *Abbreviations: SD = standard deviation; SWCT = supraventricular wide complex tachycardia; VT = ventricular tachycardia.* [↑](#footnote-ref-5)
6. Numbers in parentheses are percent (%) of n or standard deviation. *Abbreviations: ICD = implantable cardioverter-defibrillator; LVEF = left ventricular ejection fraction; SD = standard deviation; SWCT = supraventricular wide complex tachycardia; VT = ventricular tachycardia.* [↑](#footnote-ref-6)
